# Epigenetic modifications and NF-κB pathway activity in Cu,Zn-SOD-deficient mice

**DOI:** 10.1007/s11010-014-2186-0

**Published:** 2014-08-20

**Authors:** Agnieszka Siomek, Daniel Gackowski, Anna Szpila, Kamil Brzóska, Jolanta Guz, Barbara Sochanowicz, Marcin Kruszewski

**Affiliations:** 1 Department of Clinical Biochemistry, Collegium Medicum in Bydgoszcz, Nicolaus Copernicus University, ul. Karlowicza 24, 85-092 Bydgoszcz, Poland; 2 Centre of Radiobiology and Biological Dosimetry, Institute of Nuclear Chemistry and Technology, ul. Dorodna16, 03-195 Warsaw, Poland; 3 Independent Laboratory of Molecular Biology, Institute of Agricultural Medicine, ul. Jaczewskiego 2, 20-950 Lublin, Poland

**Keywords:** Cu,Zn-SOD deficiency, Epigenetic, NF-κB pathway

## Abstract

The aim of this study was to examine the possible impact of Cu,Zn-SOD deficiency on the level of epigenetic modifications in different mouse tissues, and the relationship between these modifications and the NF-κB transcription factor activity. Cu,Zn-SOD deficiency did not influence the level of 5mdC or 5hmdC in the analyzed tissues. Statistically significant organ-/tissue-specific differences between the levels of 5mdC and 5hmdC were demonstrated within each genotype. Also correlations between analyzed parameters pointed to wide tissue/genotype variety; we observed a positive correlation between 5mdC and NF-кB proteins, p50 and RelA, in the liver of wild mice, as well as an inverse correlation between 5mdC and p65 in the brain of Cu,Zn-SOD-deficient animals. Moreover, a positive correlation was revealed between 5mdC and 5hmdC in the liver and brain of knockout mice. As the highest levels of both 5mdC and 5hmdC were observed in the brains of analyzed animals regardless of their genotype, and lower, comparable to each other, levels of these modifications were shown in the kidney and liver, active demethylation process seems to be tissue-/organ-specific and does not necessarily rely solely on the redox/oxidation state of cells. According to the most likely scenario, various tissues may differ in terms of their metabolic rates, which has potential influence on cofactors, and consequently on the activity of TET enzymes or activation of TET-independent mechanisms.

## Introduction

Epigenetic mechanisms that can impact expression of genes include molecular modification of the DNA (e.g. methylation) or histones (e.g. acetylation, deacetylation, phosphorylation and methylation) [1]. Methylation of DNA is catalyzed by enzymes of DNA methyltransferase (DNMT) class, which use S-adenosyl methionine as a donor for the methyl group [2]. The most extensively characterized epigenetic mark is a methyl group at the 5-position of cytosine bases, and cytosine methylation plays an important role in key biological processes [3]. A number of recent studies have demonstrated that methylation of DNA in mammalian cells is a reversible process, and demethylation may result from both passive and active mechanisms. The active pathway includes ten-eleven translocation (TET) and involves enzymes of AID/APOBEC families [4]. As shown in early 1970s by Penn et al. [5], 5mC in mammalian DNA can be hydroxylated to 5-hydroxymethylcytosine (5hmC). Recent studies demonstrated that genomic DNA may contain approximately 0.003–0.6 % of 5hmC, referred to as “the sixth base”, and a number of experiments confirmed the pivotal role of this modification in active DNA demethylation mechanisms [4, 6, 7]. Furthermore, some authors showed that 5hmC might be also synthesized outside the TET pathway, e.g. through ultraviolet irradiation of 5mC in aerated aqueous solution [8], or can arise as a result of the addition of formaldehyde to DNA cytosine by DNMT1-mediated oxidation [9]. Moreover, the level of 5hmC was shown to be particularly high in certain tissues, such as brain and pluripotent embryonic stem cells, and turned out to be relatively lower in blood, lungs, kidneys and muscles [10]. Oxidative stress may lead to the formation of a variety of modified DNA bases, including oxidation 5mC to 5hmC. However, Kriaucionis and Tahiliani showed no correlation between the age of adult mice and the level of 5hmC [6]. The role of TET proteins in 5mC oxidation process also underlines the possible impact of the oxidative stress on the epigenetic mechanisms [3].

Aerobic organisms developed complex antioxidant defense systems protecting them against the potential detrimental effect of oxidative stress. These systems are to a large extent based on the activity of superoxide dismutases (SODs). Three main isozymes of SOD can be found in animals, with Cu,Zn-SOD (SOD1) being responsible for the majority of total activity of this enzyme. According to ample data, a deficiency in various forms of SOD promotes oxidative damage or shortened lifespan in a wide range of organisms [11]. Mice lacking SOD1 appeared normal while young, but were less able to recover from axonal injury [12] and could not successfully reproduce. Additionally, Cu,Zn-SOD-deficient mice are characterized by decreased lifespan, higher incidence of liver cancer [13], defective energy homeostasis, and greater risk of ALS-like pathologies [14]. Mice lacking SOD1 (*Sod1* ^−*/*−*)*^ are particularly sensitive to many agents known to elevate the level of oxidative stress or to heart ischemia/reperfusion [15, 16]. The impact of Cu,Zn-SOD deficiency on the oxidative damage, molecular and metabolic alterations seems to be tissue-/organ-specific, as several tissues of *Sod1* ^−*/*−^ mice showed higher levels of oxidized lipids, proteins or DNA [13]. Furthermore, our previous study documented the presence of organ-specific oxidative stress in Cu,Zn-SOD-deficient mice, and this phenomenon proved to be restricted to liver and kidneys [17]. Moreover, previous research on antioxidant mutant mouse models showed that metabolic dysfunctions, insulin resistance and modifications of glucose homeostasis are the most prevalent oxidative stress-related alterations present in these animals [18].

In our previous study [17], we revealed that tissue-specific oxidative stress in Cu,Zn-SOD-deficient mice, expressed by the level of 8-oxodG, correlated with the NF-κB activity proteins, such as p50 and p65, in a tissue- and genotype-dependent manner. NF-кB is a transcription factor, discovered in B cells by Sen and Baltimore in 1986 [19]. It may be activated in many cells by a diverse set of stimulating agents with redox regulation properties, e.g. reactive oxygen species [20]. Activity of NF-кB is regulated at various levels and is detected either in the cytoplasm or in the nucleus. While the upstream cytoplasmic regulatory factors for the NF-кB activation are quite well known, we still know little about nuclear regulation of NF-кB. As it was demonstrated in the nucleus, the RelA subunit of NF-кB undergoes a set of stimulus-coupled posttranslational modifications, including methylation [21]. A number of recent studies centered around an association between NF-κB transcription factor activity and methylation/demethylation processes. Expression of activation-induced cytidine deaminase (AID) in gastric epithelial cells infected with cagPAI-positive *H. pylori* was shown to be mediated by IκB-kinase dependent NF-κB activation pathway [22]. Furthermore, a methylation-dependent NF-кB binding to the Caudal homeobox factor 1 (CDX1) promoter was documented, and stages of hyper/hypo-methylation of the CDX1 promoter were shown to be inversely correlated with the NF-кB signaling activity along this sequence [23].

Taking into consideration all these information and wishing to provide a better insight into the aforementioned processes we decided to use mice lacking *SOD1* as a model of increased oxidative stress to determine the association between oxidative stress, DNA methylation and NF-кB protein activity. To accomplish our goal, we decided to analyze: (i) the level of 5mdC and its derivative in different murine tissues/organs, (ii) the possible impact of the Cu,Zn-SOD deficiency on DNA methylation, and (iii) the relationship between the level of 5mdC and/or 5hmdC and the NF-κB protein activity.

## Materials and methods

### Animals

A breeder pair of mice (strain B6; 129S7-Sod1^tm1Leb^), heterozygous for the *SOD1* ^tm1Leb^ **-**targeted mutation, and their progeny were provided by the Jackson Laboratory (Bar Harbor, ME). Males and females heterozygous for the non-functional *SOD1* allele (*SOD1* ^−*/*+^) were intercrossed, and their progeny were kept at 24–25 °C, in 80 % humidity with a light–dark cycle of 12 h. The mice received a standard laboratory diet (Labofeed, Kcynia, Poland) and water ad libitum. Standard laboratory diet recommended by Labofeed consisted of protein (22 %), fat (4.2 %), fiber (3.5 %) and ash (5.7 %). Moreover, the feed materials contained sharps and cereal preparations, post-extraction soya meal, potato protein, dried whey, flax meal, yeast feed and a mixture of minerals and vitamins.

Genotyping of DNA isolated from mouse tails was performed by PCR analysis according to the protocol provided by the Jackson Laboratory. We used 12-month-old mice homozygous for the non-functional *SOD1* allele (*SOD1* ^−*/*−^), heterozygous and control mice homozygous for the wild-type *SOD1* allele (*SOD1* ^+*/*+^).

All the experimental procedures involving animals were approved by the Local Bioethics Committee (decision no. 47/2012/200).

### DNA analysis

DNA isolation from tissues, hydrolysis, preparation of nuclear extracts and NF-κB DNA-binding assay were performed as described earlier [17, 24]. Briefly, DNA-binding activity of p50 and p65 proteins in nuclear extracts was assessed using NFκB p50/p65 EZ-TFA transcription factor assay (Upstate). Absorbance was measured at 450 nm in a microplate spectrophotometer. Results were normalized to absorbance/mg protein.

### UPLC–UV–MS/MS analysis of 5-hydroxymethyl-2′-deoxycytidine and 5-methyl-2′-deoxycytidine

Genuine standards of 5-hydroxymethyl-2′-deoxycytidine (5hmdC) and 5-methyl-2′-deoxycytidine (5mdC) were obtained from Berry and Associates, Inc. (Bishop Circle East Dexter, MI), 2′-deoxythymidine and 2′-deoxycytidine from Sigma-Aldrich (Poznan, Poland), and [D3]-5-hydroxymethyl-2′-deoxycytidine from Toronto Research Chemicals (Toronto, Canada).

The DNA hydrolysates were spiked with internal standard and injected into the column in amounts corresponding to 2 pmol [D_3_]-5-hmdC. Chromatographic separation was performed with a Waters ACQUITY UPLC instrument, consisting of binary gradient pump built-in vacuum degasser sample manager, column heater and PDA detector. The HPLC was operated using MassLynx 4.1 Software from Waters. A Kinetex C18 column (150 mm × 2.1 mm, 1.7 µm) and Krude Katcher Ultra 0.5 µm in-line filter were used, both from Phenomenex. The flow rate was set at 300 µl/min and the injection volume at 2 µl. Separation was accomplished by 10 min gradient elution, using a mobile phase of 0.2 % acetate and acetonitrile (1–10 % for 5 min, followed by re-equilibration with 1 % acetonitrile for 5 min). The amounts of 2′-deoxythymidine and 2′-deoxycytidine 5-methyl-2′-deoxycytidine were determined by UV detection at 280 nm with external calibration. Subsequently, mass spectrometric detection was performed using a Waters Quattro Premiere XE tandem quadrupole mass spectrometer equipped with an electrospray ionization source. Ion source parameters were as follows: capillary voltage 3.8 kV, extractor voltage 3.5 V, source temperature 120 °C, nitrogen desolvation gas flow 800 l/h, nitrogen cone gas flow 50 l/h and desolvation temperature 350 °C. Collision-induced dissociation was performed with argon 6.0 at 3 × 10^−6^ bar pressure as the collision gas. Electrospray ionization was performed in the positive ion mode. For all analytes, [M + H]^+^ was selected by the first mass filter. The instrument response was optimized by infusion of the 10 µM genuine compound dissolved in water, in the mobile phase stream, through T-connector (10 µl/min), using MassLynx 4.1 autotune feature. To obtain the highest sensitivity, the most abundant transition patterns for each compound were selected (258 → 142 and 261 → 145) and acquired with dwell time of 50 ms, with optimal cone voltage 20 V and collision energy 12 eV.

### Statistical analysis

The levels of 5mdC and 5hmdC in DNA were analyzed with U Mann–Whitney test and Wilcoxon’s matched pairs test. To estimate the correlation between the levels of methylation markers, Spearman’s correlation coefficient (*r*) was computed. The results were considered significant at *p* ≤ 0.05. All the statistical analyses were performed using Statistica 10 software (StatSoft).

## Results

### Methylation markers

The level of 5hmdC is expressed as the amount of modified moiety per 10^3^ deoxynucleotides, whereas the level of 5mdC as the percentage of the sum of 2′-deoxycytidine and 5-methyl-2′-deoxycytidine, reflecting the level of global methylation, according to the pattern:

$$[\%\,{\rm of\;cytosine\;methylation}= (5{\rm mdC}/{\rm dC} + 5{\rm mdC})*100\;\%].$$

Cu,Zn-SOD deficiency did not influence the level of 5mdC and 5hmdC. We did not found any significant genotype-specific differences in the levels of the analyzed modifications. The levels of 5mdC in livers of *SOD* ^+*/*+^ and *SOD* ^−*/*−^ animals amounted to 3.54 ± 0.13 and 3.53 ± 0.13 %, respectively. The highest levels of 5mdC, corresponding to 4.60 ± 0.67 and 4.53 ± 0.85 % in wild and *SOD* ^−*/*−^ mice, respectively, were observed in brain tissue; moderate levels of this modification were found in kidneys: 3.44 % ± 0.12 and 3.39 % ± 0.16, respectively. The analyzed tissues of each genotype differed significantly in terms of the 5mdC levels (Tables 1 and 2).

**Table 1:** The level of 5mdC and 5hmdC in tissues of wild animals (mean value ± SD)

5hmdC			5mdC		
Liver	Kidney	Brain	Liver	Kidney	Brain
1.14^1^ ± 0.11	1.10^2^ ± 0.11	2.76^1,2^ ± 0.27	3.54^1,^ ± 0.13	3.44^,2^ ± 0.12	4.60^1,2^ ± 0.67
1; p = 0.003	2; p = 0.002		1; p = 0.017	2; p = 0.011	

Furthermore, 5hmdC was detected in all the analyzed tissues. The level of this modification was the highest in neuronal (brain) tissues, amounting to 2.76 ± 0.27 per 10^3^dN and 2.65 ± 0.11 per 10^3^dN in wild type and Cu,Zn-SOD-deficient mice, respectively.

The levels 5hmdC in DNA extracted from kidneys resembled those documented in the liver (Tables 1 and 2), and amounted to 1.12 ± 0.12 per 10^3^dN. Similar to 5mdC, we documented significant genotype-specific differences in the content of 5hmC in various tissues.

**Table 2:** The level of 5mdC and 5hmdC in tissues of Cu,Zn-SOD deficient animals (mean value ± SD)

5hmdC			5mdC		
Liver	Kidney	Brain	Liver	Kidney	Brain
1.12^1^ ± 0.16	1.13^2^ ± 0.11	2.65^1,2^ ± 0.54	3.52^1,3^ ± 0.13	3.39^,2,3^ ± 0.16	4.53^1,2^ ± 0.85
1; *p* = 0.003	2 *p* = 0.003		3; *p* = 0.017	2; *p* = 0.017	1; *p* = 0.043

### NF-κB activity

Activity of p50(*NF*-*κB1)* and p65 (RelA) proteins was analyzed in nuclear extracts form the brain, kidney and liver of wild (^+/+^) and Cu,Zn-SOD-deficient mice. As we have previously reported [17], no genotype-related differences were observed in p50 or p65 DNA-binding activity in liver and brain. Significant increase in p50, but not p65, DNA-binding activity was detected in the kidneys of Cu,Zn-SOD-deficient mice as compared to the wild type animals (66.03 ± 3.04 vs. 50.33 ± 3.23).

### Correlations

**Fig. 1:**
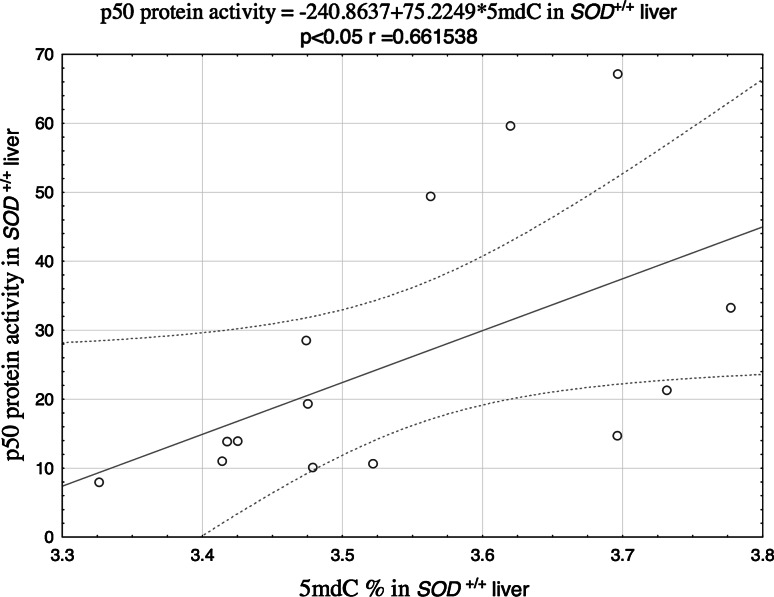
Correlation between 5mdC and p50 protein in the liver of SOD1^+*/*+^ mice

**Fig. 2:**
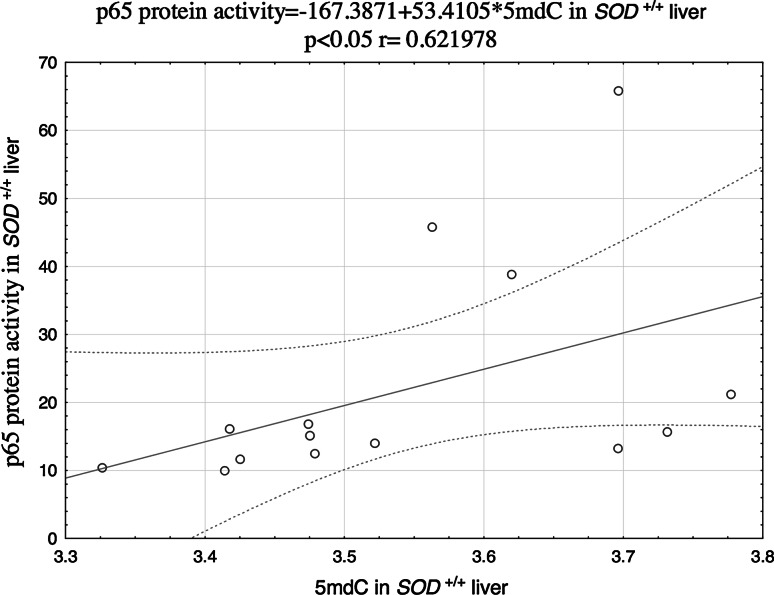
Correlation between 5mdC and p65 protein in the liver of SOD1^+*/*+^ mice

**Fig. 3:**
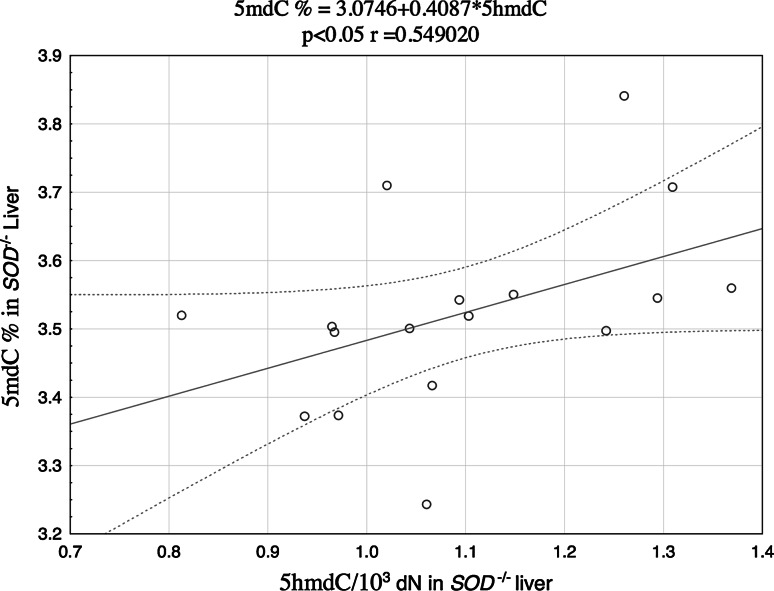
Correlation between 5mdC and 5hmdC in the liver of SOD1^−*/*−^ mice

Potential associations between the NF-κB DNA-binding activity and the levels of 5mdC and 5 hmdC in DNA were analyzed on the basis of the Spearman’s correlation coefficients (r). The relationships between the analyzed parameters turned out to be both tissue- and genotype-specific. The level of 5mdC in the liver of the wild animals showed strong positive correlation with either p50 (Fig. 1) and p65 levels (Fig. 2), and a significant correlation between 5mdC and 5hmdC levels was documented in the liver of Cu,Zn-SOD-deficient mice (Fig. 3).

**Fig. 4:**
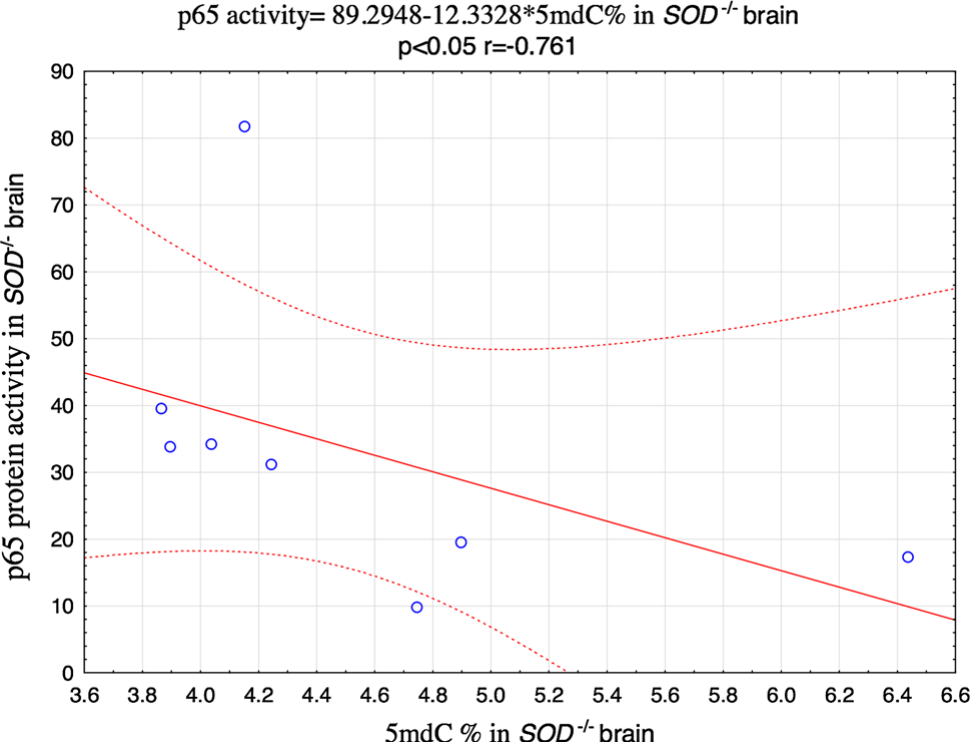
Correlation between 5mdC and p65 in the brain of SOD1^−*/*−^ mice

**Fig. 5:**
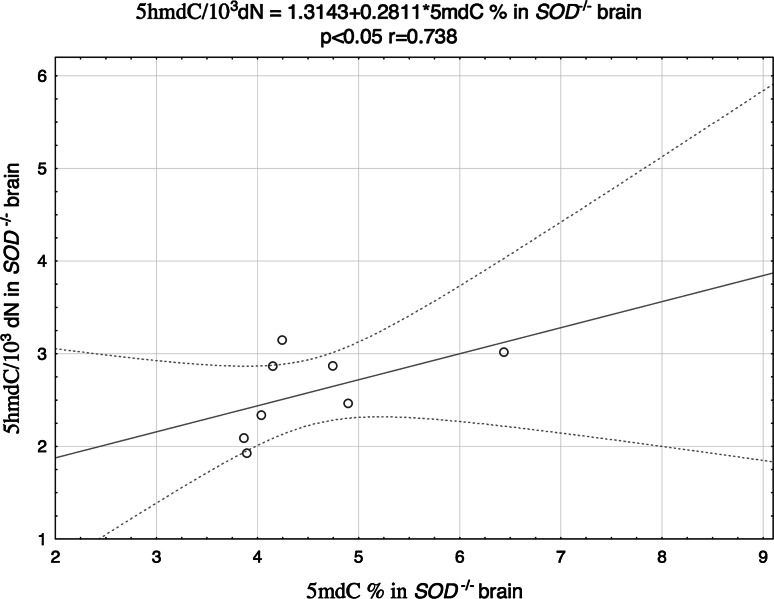
Correlation between 5mdC and 5 hmdC in the brain of SOD1^−*/*−^ mice

Furthermore, a significant inverse correlation between p65 and 5mdC was observed in the brains of Cu,Zn-SOD-deficient animals (Fig. 4), along with a positive correlation between 5mdC and 5hmdC levels (Fig. 5). No statistically significant correlations between the analyzed parameters were found in kidneys of the studied animals. Moreover, we haven’t found any associations between 8-oxodG and methylation markers in analyzed tissues (Table 3).

**Table 3:** Correlations between the level of 5mdC, 5hmdC, NF-κB proteins and 8-oxodG in analyzed tissues. Underlined values *r* are statistically significant at *p* < 0.05

	*SOD1* ^+*/*+^	*SOD1* ^−*/*−^
A The level of 5mdC in liver cells		
p50	0.66	−0.17
p65	0.62	−0.22
5hmdC	0.17	0.54
8-oxodG	0.01	−0.06
B The level of 5mdC in kidney cells		
p50	−0.17	0.19
p65	−0.11	0.08
5hmdC	−0.10	−0.01
8-oxodG	−0.03	0.27
C The level of 5mdC in brain cells		
p50	0.23	−0.69
p65	0.39	−0.76
5hmdC	−0.30	0.73
8-oxodG	−0.47	−0.11

## Discussion

Copper-zinc superoxide dismutase (Cu,Zn-SOD) is believed to play a pivotal role in the first line of antioxidant defense against reactive oxygen species. However, due to the overlapping activity of some of the antioxidant enzymes, identification of their specific roles is generally difficult. This obstacle can be overcome by the research on mice that lack a specific enzyme. Recently, the presence of 5-hydroxymethylcytosine (5hmC), formed by the oxidation of 5-methylcytosine (5mC), was discovered in various tissues and embryonic stem (ES) cells [4, 6]. Similar to 5-methylcytosine, 5-hydroxy-methylcitosine is believed to play an important role in control of gene activity. However, we still gain novel insights into 5mC/5hmC patterns and functions in mammalian cells [3, 25].

The transcription nuclear factor-κB (NF-κB) plays a prominent role in immune and stress responses, inflammation, carcinogenesis, apoptosis, cell proliferation, growth, differentiation and survival [26, 27]. Due to a link between DNA damage and NF-κB activity, understanding of molecular mechanisms underlying the control of NF-кB activation seems to be essential for the development of effective anti-inflammatory and anticancer agents. Although inhibitor IκB proteins are known as the primary control point for NF-κB activation, the NF-κB dependent gene expression can also be regulated by posttranslational modification of individual subunits of this cell signaling pathway [28].

In this study, we analyzed the influence of Cu,Zn-SOD deficiency in mammals on methylation/demethylation processes, as well as an association between epigenetic markers and activity of the NF-κB proteins. We showed that Cu,Zn-SOD deficiency does not influence the levels of 5mdC and 5hmdC. In our previous study, we found that Cu,Zn-SOD deficiency leads to organ-specific increase in the amount of oxidatively damaged DNA and p50 protein activity in mice. Oxidative stress was observed in livers and kidneys of *SOD1* ^−*/*−^ animals and was expressed by the higher level of 8-oxo-7,8-dihydro-2′-deoxyguanosine (8-oxodG) in DNA [17]. In our present study, we showed that the levels of epigenetic modifications are independent on the genotype of analyzed animals. We observed statistically significant organ-/tissue-specific differences in the levels of 5mdC and 5hmdC within each genotype. Also correlations between analyzed parameters pointed to wide tissue/genotype variety; we observed a positive correlation between 5mdC and NF-кB proteins, p50 and RelA, in the liver of wild mice, along with an inverse correlation between 5mdC and p65 in the brain of Cu,Zn-SOD-deficient animals. Moreover, a positive correlation was revealed between 5mdC and 5hmdC in the liver and brain of knockout mice.

As Cu,Zn-SOD deficiency results in higher levels of 8-oxodG in the liver and kidneys, the correlations found in our study support the observation that 5hmdC could be synthesized as a result of TET-mediated oxidation; however, the activities of this enzyme in various tissues/organs are not equal, as we did not observe oxidative stress conditions in the brain [17]. According to Liutkeviciute, in some conditions 5hmC can be formed from 5mC as a result of DNMT1-mediated reaction with formaldehyde, and “such atypical enzymatic reactions with non-cofactor-like substrates open new ways for sequence-specific derivatization of DNA and demonstrate enzymatic exchange of 5-hydroxymethyl groups on cytosine in support of an oxidative mechanism of DNA demethylation” [9]. Our findings are consistent with the results of previous studies [6], showing that the genome of an adult brain shows the highest level of 5hmC from all mammalian cell types. Moreover, our observations point to potential presence of another, additional to oxidation, source of 5hmC in the brain, perhaps utilizing the abovementioned mechanism proposed by Liutkeviciute.

Our findings are consistent with previously published data [10], demonstrating that the level of 5hmdC is tissue-/organ-specific. Moreover, we showed that the level of 5mdC correlates with the level of its hydroxylated derivative (Tables 1, 2). The wild type and knockout mice did not differ significantly in terms of their 5mdC levels in various tissues. Irrespective of the genotype, the content of 5mdC in the kidneys and liver (approximately 3 %) was markedly lower than in the brain (4.5 %). Also the brain level of 5hmdC turned to be almost three-fold higher than the content of this modification in the liver and kidneys (about 1/10^3^dN). Together with the results of our previous study, these findings suggest that active oxidative 5mC demethylation is likely cell-/line-/tissue-specific; this specificity may reflect differences in the metabolism of various tissues and resultant changes in TET protein activity. Our previous study [17] documented the presence of organ-specific oxidative stress in Cu,Zn-SOD-deficient mice, and showed that this phenomenon is limited to liver and kidneys. Our present findings suggest that Cu,Zn-SOD deficiency and resultant oxidative stress do not exert direct effect on the level of hydroxylated derivatives of 5mdC or the epigenetic marker itself. A growing body of evidence suggests that changes of metabolic status may affect the relative cellular concentration of enzymatic cofactors, thus influencing the activity of DNA and histone modifying enzymes [29].

Interesting information come from the analysis of correlation between methylation/demethylation parameters and nuclear activity of the NF-κB proteins. We found strong positive correlation between the 5mdC level and the activity of p50 and p65 proteins in wild animals. These observations may give new insight into the mechanisms underlying the interactions between NF-κB proteins and DNA. Although methylated DNA is generally perceived as a factor “blocked” for transcription, our findings suggest that some tissue-specific interaction between NF-κB proteins and methylated DNA may exist in the case of NF-κB signaling pathway. Although we did not observe the correlation between methylation/demethylation parameters and nuclear activity of the NF-κB proteins in Cu,Zn-SOD-deficient animals, we found strong positive correlations between 5mdC and 5hmdC levels in the liver and brain of these animals. This observation may support the oxidative origin of 5hmdC, since we previously showed that the liver of knockout mice is characterized by the highest level of 8-oxodG, a marker of oxidative stress. Nevertheless, the fact that we found an inverse correlation between the 5mdC level and p65 protein activity in the brain of knockout mice emphasizes the complexity of NF-κB regulation mechanisms.

The presence of methylation-dependent NF-κB binding suggests that epigenetic DNA-modification fits into the context of cell signaling pathways. However, this issue requires further research on the promoter level. Furthermore, the fact that methylation of CDX1 promoter follows a biphasic pattern with consecutive hypomethylation and hypermethylation stages emphasizes the complexity of methylation-DNA-binding mechanisms [23]. The authors of the latter study [23] documented a rare phenomenon of concomitant hyper- and hypomethylation. Therefore both their findings and our results add to existing knowledge on common regulatory mechanisms of methylation and cell signaling processes. The exact mechanisms through which DNA methylation/demethylation processes may modulate expression of genes are still not completely understood, and given the important role of NF-κB in human physiology and pathology, every new insight into this issue is valuable. Our results shine some light on these complicated mechanisms. Active demethylation process seems to be a tissue-/organ-specific and not necessarily rely solely on the redox/oxidation state of cells. According to the most likely scenario, various tissues may differ in terms of their metabolic rates, which has potential influence on cofactors, and consequently on the activity of TET enzymes or activation of TET-independent mechanisms.
